# Long Non-coding RNAs: At the Heart of Cardiac Dysfunction?

**DOI:** 10.3389/fphys.2019.00030

**Published:** 2019-01-29

**Authors:** Lisa Hobuß, Christian Bär, Thomas Thum

**Affiliations:** ^1^Institute of Molecular and Translational Therapeutic Strategies (IMTTS), Hannover Medical School, Hanover, Germany; ^2^National Heart and Lung Institute, Imperial College London, London, United Kingdom

**Keywords:** non-coding RNA, cardiac hypertrophy, myocardial infarction, cardiovascular disease, therapy

## Abstract

During the past decade numerous studies highlighted the importance of long non-coding RNAs (lncRNAs) in orchestrating cardiovascular cell signaling. Classified only by a transcript size of more than 200 nucleotides and their inability to code for proteins, lncRNAs constitute a heterogeneous group of RNA molecules with versatile functions and interaction partners, thus interfering with numerous endogenous signaling pathways. Intrinsic transcriptional regulation of lncRNAs is not only specific for different cell types or developmental stages, but may also change in response to stress factors or under pathological conditions. Regarding the heart, an increasing number of studies described the critical regulation of lncRNAs in multiple cardiac disorders, underlining their key role in the development and progression of cardiac diseases. In this review article, we will summarize functional cardiac lncRNAs with a detailed view on their molecular mode of action in pathological cardiac remodeling and myocardial infarction. In addition, we will discuss the use of circulating lncRNAs as biomarkers for prognostic and diagnostic purposes and highlight the potential of lncRNAs as a novel class of therapeutic targets for therapeutic purpose in heart diseases.

## Long Non-Coding RNAs

Although the majority of the human genome is transcribed into RNA molecules, only ∼2% of these transcripts code for proteins, thus, scientists have started to explore the wide universe of non-coding RNAs (ncRNAs) as crucial regulators of physiological and pathological cell function. Numerous studies demonstrated the involvement of small ncRNAs like microRNAs (miRNAs) in cardiovascular disease (Quiat and Olson, 2013; Thum, 2014), whereas the importance of long non-coding RNAs (lncRNAs) as key regulators in the development and progression of cardiac diseases is only beginning to be understood. As all ncRNAs longer than 200 nucleotides in length are arbitrarily classified as lncRNAs, this group of molecules is very heterogeneous and exhibit multifaceted biological functions and interact with a variety of other RNAs or proteins. Depending on their subcellular localization in the nucleus or cytoplasm, lncRNAs can interfere with transcriptional and post-transcriptional gene regulation, as well as mRNA translation, respectively. Nuclear transcripts, for example, can mediate epigenetic gene modifications or transcriptional activation and silencing, whereas cytoplasmic lncRNA often interact with miRNAs to post-transcriptionally regulate gene expression or act as molecular scaffolds for RNA-protein complexes. For further details on lncRNA modes of action we refer to the current literature available (Sun et al., 2017; Noh et al., 2018). Here, we discuss the growing body of evidence depicting a central role of lncRNAs in cellular responses during cardiac disease focusing on pathological hypertrophic cardiac growth and myocardial infarction (MI), and their potential to serve as novel therapeutic targets and diagnostic biomarkers.

## LncRNAs in Cardiovascular Disease

The term cardiovascular disease (CVD) comprises a wide range of pathologies. However, in order to adequately address the importance of lncRNAs in distinct diseases and to highlight specific molecular modes of action, we will focus on a selection of lncRNAs associated with cardiac hypertrophy and myocardial infarction. In addition, Table 1 provides an exhaustive list of relevant lncRNAs in CVD. Importantly, lncRNAs have also been shown to play essential roles in embryonic development and cardiac lineage commitment [such as Fendrr and Braveheart (Grote et al., 2013; Klattenhoff et al., 2013)] and are important regulators of accurate heart functionality. More details on lncRNAs in organogenesis and heart development have been extensively reviewed elsewhere; (Devaux et al., 2015; Grote and Herrmann, 2015).

**Table 1:** Overview of lncRNAs in different CVDs.

lncRNA	Associated disease	Reported function	Reference
Anril	Coronary artery disease	Biomarker (in diabetic type II patients)	Rahimi et al., 2018
	In-stent restenosis	Biomarker	Wang et al., 2017
	Left ventricular dysfunction	Biomarker	Vausort et al., 2014
Braveheart	Cardiac lineage development	Regulation of chromatin modifications via PRC2	Klattenhoff et al., 2013
Carl	Myocardial infarction	Inhibition of mitochondrial fission and cardiomyocyte apoptosis by sponging miR-539	Wang et al., 2014b
Chaer	Cardiac hypertrophy	Epigenetic modulation of hypertrophic gene expression	Wang et al., 2016
Chast	Cardiac hypertrophy	Induction of hypertrophic cell growth and gene expression	Viereck et al., 2016
Chrf	Cardiac hypertrophy	Sponging of miR-489	Wang et al., 2014a
	Doxorubicin-induced heart failure	Regulation of TGF-β signaling	Chen et al., 2018
Fendrr	Organ development derived from lateral mesoderm	Regulating chromatin modifications via PRC2 and TrxG/MLL	Grote et al., 2013
Ftx	Myocardial infarction	Regulation of cardiomyocyte apoptosis by targeting miR-29b-1-5p	Long et al., 2018
H19	Cardiac hypertrophy	Targeting Ca/calmodulin-dependent protein kinase IIδ (CaMKIIδ)	Liu et al., 2016
	Coronary artery disease	Biomarker	Zhang et al., 2017
	Diabetic cardiomyopathy	Regulation of cardiomyocyte apoptosis by targeting VDAC1	Li et al., 2016b
	Ischemia reperfusion injury	Regulation of necrosis by targeting miR-103/107	Wang et al., 2015
	Myocardial infarction	Activation of autophagy	Zhou et al., 2018
Hotair	Cardiac hypertrophy	Interaction with miR-19	Lai et al., 2017
Lipcar	Cardiac remodeling and heart failure	Biomarker	Kumarswamy et al., 2014
	Coronary artery disease	Biomarker	Zhang et al., 2017
	Left ventricular diastolic function	Biomarker	De Gonzalo-Calvo et al., 2016
Malat1	Atherosclerosis	Regulation of inflammation	Gast et al., 2018
	Cardiac fibrosis after myocardial infarction	Regulation of TGF-β signaling via miR-145	Huang et al., 2018
Mdrl	Ischemia-reperfusion injury	Inhibition of mitochondrial fission and cardiomyocyte apoptosis by sponging miR-361	Wang et al., 2014c
Meg3	Cardiac fibrosis and diastolic dysfunction	Regulation of TGF-β I induced p53 signaling	Piccoli et al., 2017
	Myocardial infarction	Regulation of cardiomyocyte apoptosis	Wu et al., 2018
Mhrt	Cardiac hypertrophy	Regulation of isoform switch Myh6 to Myh7	Han et al., 2014
	Doxorubicin-induced cardiomyopathy	Inhibition of cardiomyocyte apoptosis	Li et al., 2016a
	Heart failure	Biomarker	Xuan et al., 2017
Miat	Cardiac fibrosis after myocardial infarction	Regulation of cardiac fibrosis by interaction with several miRNAs	Qu et al., 2017
	Cardiac hypertrophy	Sponging miR-93	Li et al., 2018
		Sponging miR-150	Zhu et al., 2016
	Left ventricular diastolic function	Biomarker	De Gonzalo-Calvo et al., 2016
	Diabetic cardiomyopathy	Regulation of myocardial hypertrophy and apoptosis	Zhou et al., 2017
	Myocardial infarction	SNP in exon 5 as risk allele for MI	Ishii et al., 2006
Mirt1	Myocardial infarction	Suppression of NF-κB signaling	Li et al., 2017
Nron	Heart failure	Biomarker	Xuan et al., 2017
Sencr	Left ventricular diastolic function	Biomarker	De Gonzalo-Calvo et al., 2016
Wisper	Cardiac fibrosis after myocardial infarction	Alternative splicing of Plod2 mRNA by enabling nuclear localization of TIAR	Micheletti et al., 2017

### Cardiac Hypertrophy

One of the first studies on functionally relevant lncRNAs in cardiac hypertrophy discovered a cluster of lncRNAs partially overlapping the Myh7 gene locus which, accordingly, were called *myosin heavy-chain-associated RNA transcripts* (Mhrt) (Han et al., 2014). Mhrt was permanently downregulated after induction of cardiac hypertrophy by transverse aortic construction (TAC) surgery in mice. In addition, the dynamic regulation of this conserved, cardiac-specific lncRNA was accompanied by the TAC-induced isoform switch from Myh6 to Mhy7, a hallmark of developing cardiomyopathy (Miyata et al., 2000; Krenz and Robbins, 2004). Inducible transgenic overexpression of Mhrt resulted in reduced cardiac hypertrophy and fibrosis and improved cardiac function compared to TAC operated mice without reactivated Mhrt (Han et al., 2014). Importantly, this effect was observed when Mhrt expression was induced before TAC surgery as well as 2 weeks after pressure overload initiation, indicating that downregulation of Mhrt is important for the progression of pressure overload induced cardiac remodeling. Mechanistically, Mhrt directly interacts with the chromatin-remodeling factor Brg1 in order to inhibit its own transcriptional silencing at the shared Mhrt/Myh6 bidirectional promoter region under physiological conditions. In contrast, during cardiac stress Brg1 expression exceeds Mhrt abundance, resulting in active Brg1-mediated chromatin remodeling than leads to Mhy6 to Mhy7 isoform switch. This thereby represents an important regulatory circuit in the development and progression of cardiac hypertrophy.

Using a microarray approach to compare the lncRNA transcriptome of TAC versus sham operated mice, Viereck and colleagues identified the conserved lncRNA *cardiac hypertrophy-associated transcript* (Chast) to be upregulated in hypertrophic cardiomyocytes (Viereck et al., 2016). Chast expression is, at least partially, induced via the pro-hypertrophic transcription factor nuclear factor of activated T cells (NFAT) and acts in cis to regulate Pleckstrin homology domain–containing protein family M member 1 (Plekhm1), resulting in impaired autophagy. Remarkably, adeno-associated virus (AAV)-overexpression of Chast was sufficient to induce hypertrophic growth *in vitro* and *in vivo* in the absence of additional hypertrophic stress factors. In contrast, silencing of Chast using GapmeR antisense chemistries (for further details see section below ‘LncRNAs as potential therapeutic targets in CVD’) prevented hypertrophic cardiac growth and preserved cardiac function in TAC operated animals. Of note, silencing Chast was cardio-protective in a preventive approach, as well as in a clinically more relevant therapeutic approach with repeated GapmeR injection starting 4 weeks after induction of pressure overload in mice. Strikingly, the human CHAST transcript was able to induce hypertrophic cell growth in murine cardiomyocytes *in vitro*, suggesting functional conservation. Furthermore, CHAST was upregulated in hearts of patients with aortic stenosis, where cardiac hypertrophy occurs as a compensatory response to increased afterload, highlighting the therapeutic potential of CHAST for the treatment of cardiac hypertrophy in humans.

Another example of a pro-hypertrophic lncRNA, which emphasizes the importance of lncRNAs to precisely regulate a switch in gene expression upon cardiac stress, is *cardiac-hypertrophy-associated epigenetic regulator* (Chaer). Following TAC surgery Chaer-knockout mice showed less hypertrophic cardiac growth, reduced fibrosis and preserved cardiac function in comparison to wildtype control animals (Wang et al., 2016). In contrast, overexpression of Chaer induced hypertrophic cell growth in both phenylephrine and vehicle treated cardiomyocytes. The mainly nuclear located Chaer directly interacts with the EZH2 subunit of polycomb repressive complex 2 (PRC2), resulting in reduced H3K27 trimethylation at promoter regions and thereby enhanced expression of the pro-hypertrophic genes Anf, Myh7, and Acta. Furthermore, the authors highlighted the pivotal role of Chaer-PCR2 interaction at the onset of pathological cardiac stress by knocking down Chaer expression either 2 days before or 1 day after TAC surgery. Loss of Chaer at the very beginning of pathological pressure overload reduced hypertrophic heart growth and marker gene expression and improved cardiac function compared to control animals, while Chaer knockdown 24 h post TAC showed no protective effect. This early interaction between PRC2 and Chaer seems to be required for the onset of cardiac epigenetic reprogramming but not progression of hypertrophic remodeling.

**Figure 1:**
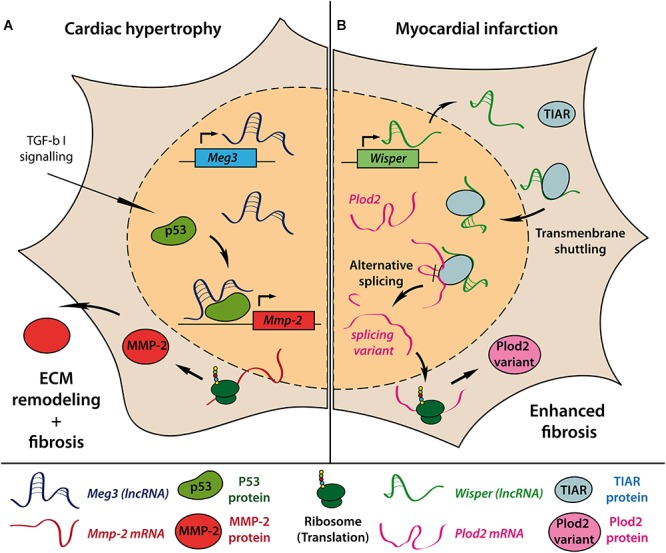
Molecular mechanisms of lncRNAs involved in cardiac fibrosis. Introduction of pressure-overload-induced cardiac hypertrophy in mice causes upregulation of the lncRNA Meg3 in cardiac fibroblasts. Interaction of Meg3 with the transcription factor p53 is required for TGF-β–induced transcription of *Mmp-2* gene. Increased extracellular MMP-2 levels subsequently drive enhanced extracellular matrix (ECM) remodeling and cardiac fibrosis **(A)**. Myocardial infarction causes upregulation of lncRNA Wisper in cardiac fibroblasts. Only in the presence of Wisper can TIAR shuttle from the cytoplasm to the nucleus to interfere with RNA processing and induce alternative splicing of Plod2 mRNA. The resulting alternative Plod2 protein variant causes enhanced fibrosis **(B)**.

Besides hypertrophic growth of cardiomyocytes, pressure overload induced pathological remodeling is accompanied by cardiac fibroblast (CF) activation and rearrangement of the extra cellular matrix (ECM), resulting in fibrosis and impaired cardiac function. By performing lncRNA array analysis in CFs of mice undergoing 13 weeks of TAC, Piccoli et al. identified over 1400 deregulated lncRNAs in CF fractions of hypertrophic mouse hearts (Piccoli et al., 2017). The most abundant and CF-specific lncRNA *Maternally expressed gene 3* (Meg3) appeared to be upregulated during the first 4 weeks after TAC followed by a long term repression. Meg3 interaction with p53 facilitates transcriptional activation of matrix metalloproteinase-2 (MMP-2) and subsequent remodeling of ECM composition (Figure 1A). Consecutive injection of GapmeRs targeting Meg3 reduced cardiac fibrosis and ameliorated cardiac performance and diastolic function in TAC operated mice compared to control GapmeR treated animals. Interestingly, knockdown of Meg3 also diminished hypertrophic cardiomyocyte growth, although Meg3 showed no TAC-induced regulation in cardiomyocytes in the initial screen suggesting further paracrine effects of Meg3 silencing in CFs on other cardiac cell types.

### Myocardial Infarction

The lncRNA *myocardial infarction associated transcript* (MIAT) was first described in a large scale single nucleotide polymorphism (SNP) association study from a Japanese population in 2006. In this study the presence of six SNPs in the MIAT locus was associated with an increased risk for MI (Ishii et al., 2006). *In 2017*, Qu and colleagues also identified MIAT as a pro-fibrotic lncRNA after MI *in vivo* (Qu et al., 2017). CF activation and differentiation, cardiac fibrosis and scar formation represent key events after MI. On the one hand, fibrotic remodeling of the infarct region is crucial for sustaining myocardial integrity and preventing cardiac wall rupture during wound healing processes. On the other hand, sustained fibrosis throughout subsequent cardiac remodeling contributes to increased cardiac stiffness, impaired cardiac function and development of heart failure (Van Den Borne et al., 2010). In regards to MIAT, permanent occlusion of the left anterior descending artery (LAD) in mice resulted in it’s continuously upregulated expression in peri-infarcted tissue (Qu et al., 2017). By targeting several anti-fibrotic miRNAs (including miR-24, miR-29, miR-30, and miR-133), MIAT was shown to promote cardiac fibrosis and adverse remodeling in the infarcted mouse hearts. In contrast, lentiviral-mediated knockdown of MIAT prior to MI reduced infarct size and interstitial fibrosis contributing to preserved cardiac function, via the control of CF proliferation and collagen production. Additionally, two other publications highlighted further potential of MIAT to act as a pro-hypertrophic lncRNA in cardiomyocytes by sponging the anti-hypertrophic miR-150 (Zhu et al., 2016) and miR-93 (Li et al., 2018). Altogether, these findings emphasize the complex regulatory network of MIAT during cardiac disease and strengthen its potential to serve as a therapeutic target.

Recently, the conserved lncRNA *Wisp2 super-enhancer–associated RNA* (Wisper) has been described to control CF functions *in vitro* and *in vivo*. Wisper is transcribed from a cardiac-specific super-enhancer region and was found to be highly upregulated in the border zone after MI in mice (Micheletti et al., 2017). Wisper regulated proliferation, migration, and apoptosis as well as gene expression of pro-fibrotic factors like Col3a1, Fn1, and Tgfb2 in CFs but not fibroblasts from other origin. *In vivo*, GapmeR-induced knockdown of Wisper after MI resulted in a smaller infarct size, reduced fibrosis and preserved cardiac structure and function. Of note, pre-operative GapmeR treatment also impaired CF function in the acute wound healing process leading to increased mortality due to left ventricular wall rupture. The authors demonstrated that Wisper directly interacts with TIA1-related protein (TIAR) to regulate alternative splicing of lysyl hydroxylase 2 (LH2 or Plod2), which has been associated with fibrosis-related disorders (Yeowell et al., 2009; Figure 1B). Knockdown of Wisper suppressed transmembrane shuttling of TIAR protein to the nucleus and prevented TIAR-Plod2-mRNA interaction. Consequently, reduced Plod2 expression coincided with the described phenotypical changes in CF function *in vitro* and *in vivo*.

During MI, oxygen-deficiency primarily serves to induce massive loss in viable cardiomyocytes by apoptotic and necrotic cell death. Several studies have highlighted the regulatory involvement of lncRNAs in apoptotic cell death during MI. For example, the lncRNAs *cardiac apoptosis-related lncRNA* (Carl) (Wang et al., 2014b) and *mitochondrial dynamic related lncRNA* (Mdrl) (Wang et al., 2014c) were downregulated after MI. Adenoviral overexpression of Carl or Mdrl was able to inhibit mitochondrial fission and cardiomyocyte apoptosis by inhibiting pro-apoptotic miRNAs miR-539 or miR-361, respectively, and resulted in smaller infarct sizes *in vivo*. Similar to Carl and Mdrl, the lncRNA *five prime to Xist* (Ftx) is transcriptionally repressed after ischemia reperfusion injury and showed anti-apoptotic potential by regulating the Bcl2l2 repressor miRNA miR-29b-1-5p (Long et al., 2018) *in vitro*.

**Figure 2:**
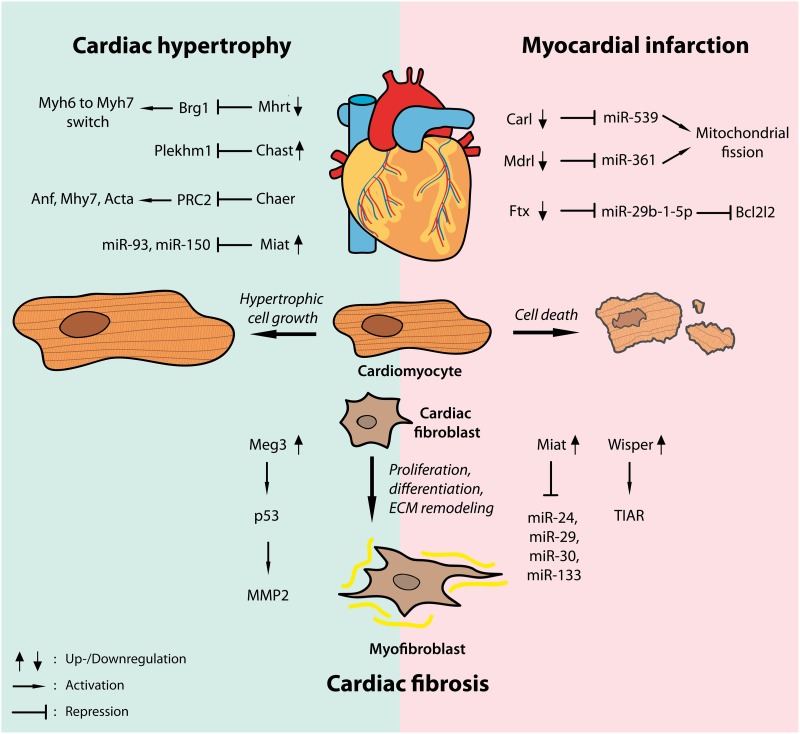
Widespread regulatory functions of lncRNAs in cardiac disease. Dynamic regulation of a number of lncRNAs has been shown in models of cardiac hypertrophy (left, blue shade) as well as myocardial infarction (right, pink shade) driving hypertrophic cardiomyocyte growth and cardiac fibrosis or regulating cardiac cell death by interfering with transcription factors, epigenetic modifiers or different microRNAs.

As highlighted above lncRNAs are crucial regulators of pathological cardiomyocyte growth, fibrosis and cell survival in hypertrophic and infarcted hearts (Figure 2). Through interfering with epigenetic gene regulation, transcriptional activation or repression and post-transcriptional modifications by directly interacting with proteins or other ncRNAs such as miRNAs, lncRNAs orchestrate miscellaneous intracellular signaling pathways in a number of cardiac diseases.

Besides intracellular signaling, changes in lncRNA expression levels also have the potential to influence intercellular communication by modulating paracrine signaling. The lncRNA *myocardial infarction-associated transcript 1* (Mirt1) for example was shown to be involved in the regulation of the acute inflammatory response after MI (Li et al., 2017). In their study Li and colleagues demonstrated that hypoxia-induced upregulation of Mirt1 in neonatal mouse CFs facilitated nuclear transport of NF-κB and expression of the pro-inflammatory cytokines IL-6, IL-1β, and TNF-α. Secretion of these pro-inflammatory cytokines in turn mediated enhanced cardiomyocyte apoptosis and macrophage infiltration into the infarcted tissue.

In addition to the aforementioned modulation of paracrine signaling, first studies have appeared describing that secreted lncRNA display a novel layer of intercellular communication themselves by mediating cell proliferation (Zhang et al., 2018) or cancer progression (Pan et al., 2017). Furthermore, circulating lncRNAs might also have the potential to act in an endocrine manner and provoke systemic responses in CVD. Regardless, further studies need to be performed to reveal the consequences of altered lncRNA levels in the systemic circulation.

## LncRNA as Biomarkers

As outlined above, lncRNAs have multifaceted intracellular regulatory functions and the capability to directly or indirectly alter intercellular communication. Furthermore, it has been shown that lncRNAs can be detected in extracellular body fluids such as plasma or urine and display a dynamic alteration upon diseases (Zhou et al., 2015; Martignano et al., 2017; Terracciano et al., 2017). LncRNAs can enter the blood stream encapsulated in exosomes (Li et al., 2015) and extracellular vesicles or inside of apoptotic bodies released from dying cells (Viereck and Thum, 2017). Beside this, association with RNA-binding proteins might also explain the enhanced stability of circulating lncRNAs conferred by their resistance to rapid degradation by RNases. Long term stability of lncRNAs in easily accessible body fluids, in combination with disease specific abundancy patterns, make lncRNAs of particular interest as a novel class of non-invasive prognostic and diagnostic biomarker.

One prominent example of lncRNAs as a potential biomarker is the mitochondria-derived lncRNA *long intergenic non-coding RNA predicting cardiac remodeling* (LIPCAR), whose plasma levels are associated with left ventricular (LV) remodeling after MI and increased risk of developing heart failure (Kumarswamy et al., 2014). Kumarswamy et al. reported a higher abundance of LIPCAR in the plasma of patients with consecutive heart failure after MI compared to MI patients without LV remodeling. Furthermore, the magnitude of circulating LIPCAR was also associated with an increased risk of cardiovascular death in chronic heart failure patients. In a 2-year follow-up, circulating LIPCAR was further identified as an independent predictor for diastolic dysfunction in well-controlled type 2 diabetes patients (De Gonzalo-Calvo et al., 2016). In addition, two other circulating lncRNAs, MIAT and *smooth muscle and endothelial cell-enriched migration/differentiation-associated long non-coding RNA* (SENCR), were associated with LV cardiac remodeling in these patients from the same study. Moreover, Zhang et al. reported increased plasma levels of LIPCAR and the paternally-imprinted lncRNA H19 in patients with coronary artery disease (CAD), especially in those subjects with concomitant chronic heart failure (Zhang et al., 2017).

Other recent studies identified the circulating lncRNAs *non-coding repressor of NFAT* (NRON) and MHRT as further independent predictors for heart failure (Xuan et al., 2017) and associated increased plasma levels of ANRIL with a higher risk for in-stent restenosis (Wang et al., 2017).

The possibility of detecting lncRNAs in extracellular body fluids discloses an enormous pool of molecules to extend the current catalog of prognostic and diagnostic biomarkers for cardiovascular and other diseases (Gutschner et al., 2017; Viereck and Thum, 2017).

## LncRNAs as Potential Therapeutic Targets in CVD

CVDs still represent the number one cause of death worldwide (World Health Organization, 2017). As highlighted above, cardiac-specific *in vivo* modulation of lncRNAs exhibit the potential to ameliorate cardiac dysfunction or diminish pathological progression in the diseased heart (Han et al., 2014; Viereck et al., 2016; Wang et al., 2016; Micheletti et al., 2017; Piccoli et al., 2017; Qu et al., 2017), potentially making them new targets for the treatment of CVDs. LncRNAs may represent therapeutic targets, provided their expression can be modulated *in vivo*.

Silencing of RNA molecules can be achieved by the use of sequence-specific antisense oligonucleotides (ASO) or RNA interference (RNAi) methods. Antisense drug therapies have already been applied in clinical trials targeting protein-coding mRNAs with one compound already on the market for the treatment of familial hypercholesterolemia [Mipomersen (Rader and Kastelein, 2014; Geary et al., 2015; Santos et al., 2015)] and another approved by the Food and Drug Administration to treat Duchenne muscular dystrophy [Eteplirsen (Mendell et al., 2013, 2016)]. In contrast to protein-coding mRNAs, lncRNAs may exert different functions with respect to their subcellular localization (nucleus or cytoplasm). This needs to be considered for the general targeting strategy. SiRNAs, for example, mainly function in the cytoplasm, therefore may be less effective against nuclear localized lncRNAs (Lennox and Behlke, 2016). In addition to subcellular localization, tissue and/or cell type-specific delivery of antisense therapeutics is crucial for targeted lncRNA modulation in different CVDs. Different delivery strategies to improve targeting of the heart have been reviewed by Lucas and colleagues (Lucas et al., 2018).

Currently, GapmeRs are the most promising class of ASOs used for pharmacological silencing of lncRNAs *in vivo*, as they are able to enter the nucleus, thus, enable targeting of nuclear transcripts as well. GapmeRs consist of a DNA core flanked by two locked nucleic acids (LNA) sequences complementary to the target mRNA or lncRNA sequence. By chemically ‘locking’ the ribose backbone of the nucleotide structure, LNAs display a higher stability, target specificity and RNase H activation potential resulting in enhanced knockdown efficiency (Swayze et al., 2007). To date, no clinical trials targeting lncRNAs have been performed. This might be due to the relative novelty of lncRNAs been regarded as potential therapeutic targets compared to proteins. However, therapeutic GapmeR injections have successfully been used to modulate lncRNAs in animal models of pressure overload [Chast (Viereck et al., 2016) and Meg3 (Piccoli et al., 2017)] and MI [Wisper (Micheletti et al., 2017)]. In all of the mentioned studies the authors presented remarkably improved cardiac function upon therapeutic intervention, stressing the great potential of antisense drugs therapeutically targeting lncRNAs. Nevertheless, the use of LNA oligonucleotides may be associated with hepatotoxicity (Swayze et al., 2007; Burdick et al., 2014), highlighting the need for further chemical refinement of this novel class of drugs. For detailed information on the pharmacology of antisense drugs, different modification strategies, and current clinical trials in general and CVDs we refer to further literature specifically discussing these topics in depth (Bennett et al., 2017; Lucas et al., 2018; Smith and Zain, 2019).

In contrast to silencing approaches, therapeutic overexpression of lncRNAs *in vivo* appears to be more challenging and requires the use of viral-mediated gene delivery, nanoparticles, or RNA mimics. Overexpression of lncRNAs using viral gene delivery poses several obstacles including the efficiency of lncRNA upregulation itself. The second challenge is to overexpress the target lncRNA in its endogenous subcellular localization – in other words, to enhance the function of cis regulatory lncRNAs via ectopic overexpression. AAV vectors that are commonly used for gene therapy approaches have relatively low packaging limit. Hence, they cannot be used for lncRNA transcripts longer than 3–4 kb. Furthermore, depending on the pathological context, a transient or stable overexpression may be needed for the therapeutic treatment. Of note, the targeted lncRNA should be overexpressed in a cell type specific manner, as lncRNAs may have varied functions in different cells or organs (Raveh et al., 2015). Despite the aforementioned shortcomings, two independent studies provided proof of principle for cardio protective viral-based overexpression strategies in a preventive therapy approach using the MI mouse model (Wang et al., 2014b,c). Although a pre-MI treatment does not represent a clinically relevant scenario, these two studies provide first evidence for the prospective potential of lncRNA overexpression as a promising therapeutic intervention in cardiovascular diseases.

In summary, a vast number of lncRNAs are dynamically regulated upon initiation and progression of CVDs. Many have important biological functions and/or have the potential to serve as a novel class of circulating biomarkers. Several *in vivo* experiments have revealed that modulation of lncRNAs offers a promising new therapeutic approach to treat cardiovascular diseases, albeit the silencing or overexpression approaches still require further refinements. Nevertheless, large screening approaches are often performed in animal models of CVD and lncRNAs are not always conserved among species but this is a prerequisite for clinical translation. However, as the field of lncRNAs as potential therapeutic targets is still in its infancy it is not unlikely that in the near future lncRNAs will emerge as valuable new tools for the treatment of numerous diseases, including CVDs.

## Author Contributions

LH wrote the manuscript and prepared the display items. CB and TT guided the manuscript preparation and critically revised it.

## Conflict of Interest Statement

TT has filed patents about the diagnostic and therapeutic use of several cardiovascular lncRNAs and is a founder of and holds shares in Cardior Pharmaceuticals. The remaining authors declare that the research was conducted in the absence of any commercial or financial relationships that could be construed as a potential conflict of interest.
